# COGNITIVE CHANGE AMONG NURSING HOME RESIDENTS; COGRISK-NH SCALE DEVELOPMENT TO PREDICT DECLINE

**DOI:** 10.1093/geroni/igad104.2286

**Published:** 2023-12-21

**Authors:** Elizabeth Howard, John Morris

**Affiliations:** Boston College Connell School of Nursing, Boston, Massachusetts, United States; Hebrew SeniorLife, The Marcus Institute for Aging Research, Boston, Massachusetts, United States

## Abstract

**Objectives:**

Chronicle cognitive changes in nursing home residents; develop risk model identifying predictors of decline.

**Methods:**

Using secondary analysis design with MDS data, cognitive status and change measures were calculated based on Cognitive Performance Scale (CPS). Baseline and quarterly follow-up analyses of US and Canadian interRAI data (n=1,257,832) were completed. Risk model from logistic regression analyses identified predictors of decline.

**Results:**

Baseline 15% of residents were cognitively intact (CPS = 0); 11.2% borderline intact (CPS=1), 15% mild impairment (CPS = 2). 58.8% of residents fell into more severe CPS categories (3-6). Over time, increased proportion of residents declined – 17.1% at 6 months, 21.6% at 9 months, at 21 months, 34.0%. Over same time, more residents remained stable than declined. Baseline CPS score was strong predictor of decline. CPS categories 0-2 had 3-month decline rates in mid-teens, categories 3-5 had average decline rate of 9%. Two strata risk model construction was employed – one for CPS categories 0-2, second categories 3-5 and both were integrated into 6-category risk scale (CogRisk-NH). Mean decline rates at 3-month assessment ranged from 4.4% to 28.3%. Over time, distinction among risk categories continued – 6.9% to 38.4.% at 6 months, 16.2% to 61.4% at 21 months. Case distribution had 15.9% in category 1, 26.84% category 2, and 36.7% category 3. Three higher risk categories (4-6) represented 20.6% of residents.

**Conclusion:**

CogRisk-NH scale differentiates among residents and likelihood of decline. Knowledge of risk for cognitive decline enables allocation of resources targeting amenable factors contributing to decline.

